# Acoustic Emission Characteristics and Change the Transformation Entropy after Stress-Induced Martensite Stabilization in Shape Memory Ni_53_Mn_25_Ga_22_ Single Crystal

**DOI:** 10.3390/ma13092174

**Published:** 2020-05-08

**Authors:** László Zoltán Tóth, Lajos Daróczi, Elena Panchenko, Yuri Chumlyakov, Dezső László Beke

**Affiliations:** 1 Department of Solid State Physics, University of Debrecen. P. O. Box 400, H-4002 Debrecen, Hungary; toth.laszlo@science.unideb.hu (L.Z.T.); lajos.daroczi@science.unideb.hu (L.D.); 2 Siberian Physical Technical Institute, Tomsk State University, Tomsk 634050, Russia; panchenko@mail.tsu.ru (E.P.); chum@phys.tsu.ru (Y.C.)

**Keywords:** stress-induced martensite aging, shape memory alloys, acoustic emission, transformation entropy

## Abstract

Measurements have been carried out to compare stress-induced martensite stabilization aged (SIM-aged) and as grown shape memory Ni_53_Mn_25_Ga_22_ single crystals with the means of simultaneous differential scanning calorimetry (DSC) and acoustic emission (AE). Contrary to expectations, the position of the hysteresis practically did not change, whilst the width of the hysteresis increased, and the forward and reverse transitions became sharper in the SIM-aged sample. The energy distributions of acoustic hits showed regular power law behaviour and the energy exponents were slightly different for heating and cooling; this asymmetry had different signs for the SIM-aged and as grown samples. During heating, in accordance with the sharper transitions observed in the DSC runs, two well-marked jumps could be seen on the plot of cumulative number of the acoustic emission events. Therefore, these were attributed to high sudden jumps in the phase transition during heating observed in the DSC. The effect of the SIM-aging on the transformation entropy was also investigated and it was found that it was about 36% less in the case of the SIM-aged sample.

## 1. Introduction

Martensite stabilization heat treatments under uniaxial stress, large enough to produce a single variant martensite structure, can result in stabilization of this martensite variant [1,2,3,4]. Such annealing, carried out at well-defined temperatures allowing atomic diffusion, is called stress-induced martensite stabilization aging or SIM-aging, (see, e.g., [5,6,7]). This stabilization eventuates improved shape memory properties, like high experimental values of reversible strains (close to the theoretical limits) and stabilization of the two-way shape memory. For instance, it was shown in [5] that SIM-aging can result in large reversible two-way shape memory behaviour in Ni_2_MnGa single crystal. Thus, in order to reach high-deformation strains (close to the theoretical limits), due to the shift of the transformation temperatures to higher values and the stabilization of the two-way shape memory properties, high-temperature, high-strength ferromagnetic shape memory alloys were developed [1,4,5,6,7]. In addition, SIM-aging can also result not only in two-way shape memory behaviour (with a reversible tensile strain of 9%), but giant rubber-like behaviour was also observed up to 15%, during reorientation of martensite variants [7]. This exceeded, by about a factor of two, the maximum compression transformation strain during the B2-L1_0_ martensitic transformation. Furthermore, it can also enhance the thermal cycling stability [8]. Thus, SIM-aging is an attractive method for development of different actuators [1,2,6,8,9].

The main features of the above stabilization can be understood on the basis of the so-called symmetry conforming short range ordering process [4,10,11,12]. While this plausibly predicts the shift of transformation temperatures, *T_o_*, to higher values, the SIM-aging, by giving preference to one martensite variant, should have important additional effects (related to dissipative and elastic energies) on the martensitic transformation. It was observed that the SIM-aging caused not only the shift of *T_o_* [1,5], but the transitions were also sharpened and even burst-like transitions were observed [5,6,7,13,14]. In our previous paper [15], in accordance with the suggestion of Kustov et al. [16], we published detailed investigations on both the shift and width of the hysteresis (which are related to the shift of *T_o_* and the dissipative energies, respectively) and on the change of broadening of the transition in shape memory Ni_51_Fe_18_Ga_27_Co_4_ single crystals. In addition, in [15] the differential scanning calorimetry (DSC) measurements were complemented using simultaneous acoustic emission (AE) measurements, and it was demonstrated that the sharp transitions produced increased numbers of acoustic emission events. It was also shown that during heating a few high-energy solitary events appeared, which were attributed to high sudden jumps in the phase transition. Thus AE investigations can provide interesting details about the jerky character of the structural changes during martensitic transformations (see also [17,18,19,20]).

Therefore, in the present paper we report comparative investigations of the martensitic transformation in SIM-aged and as grown (not SIM-aged) shape memory Ni_50_Mn_28.5_Ga_22_ single crystals using DSC and AE measurements. These results, together with the results obtained in [15] will provide a good set of experimental data for better understanding of the effects of SIM-aging. This is the first time that after the recent first reports on the SIM-aging in this alloy [5,21,22] results of acoustic emission measurements have also been published.

Finally, it should be added that there is a long-standing debate on the effect of martensite stabilization treatments on the transformation entropy. In the literature it was quite widely accepted, on the basis of the arguments published in [10,16,23], that the entropy change caused by the stabilization of the martensite or austenite is negligible. On the contrary, in an earlier paper [24] it had already been concluded that such an assumption would be improper. Indeed, we found in Ni_51_Fe_18_Ga_27_Co_4_ single crystals [15] that the transformation entropy decreased by about 36% after SIM-aging and it was practically unchanged after austenite stabilization treatment. Although the signs of the changes in our previous results [15] were in accordance with the data given in [24], we observed about a three times larger decrease due to SIM-aging. This fact also calls for further measurements and thus we investigated the change of the transformation entropy in the Ni_53_Mn_25_Ga_22_ alloy too.

## 2. Experimental Procedures

An ingot of the Ni_53_Mn_25_Ga_22_ (at %) alloy was prepared using vacuum induction melting. The single crystals were grown using the Bridgman method in a helium atmosphere. The raw samples had dimensions of 3 mm × 3 mm × 6 mm. The samples were annealed at 1273 K for 1 h and then slowly cooled to room temperature, which resulted in L2_1_ chemically ordered austenite crystal structure (these samples are called “as grown” below).

The SIM-aging was performed using an Instron VHS 5968 testing machine (Instron, Norwood, MA, USA) with a nominal 1 × 10^−3^ s^−1^ strain rate [6]. The sample underwent a compressive stress (along the [110]_L21_ direction) induced forward martensitic transformation at 423 K and it was followed by heat treatment at this temperature, under 175 MPa compressive stress, for 2 h along the [110]_L21_||[100]_L10_ direction (SIM-aged sample). These SIM-aged crystals undergo martensitic transformation to non-modulated tetragonal L1_0_ martensite [5,22].

The calorimetric measurements and their evaluations were similar to the usual procedure described in [25,26,27,28]. The masses of the examined as grown as well as SIM-aged samples were 44.3 mg and 14.2 mg, respectively. The DSC measurements were carried out with a Perkin Elmer DSC 7 device (PerkinElmer Inc., Waltham, MA, USA), using 10 K/min driving rate for the as grown and, due to the sharper transition, 5 K/min for the SIM-aged sample. The background noise was filtered and the calibration was made with the melting point of pure tin as well as pure indium.

The acoustic emission measurements were carried out in the DSC device using 10 K/min driving rate for the as grown and 1 K/min for the SIM-aged sample in order to avoid the overlapping of AE events due to the intensified transformation. Sensophone AED 404 Acoustic Emission Diagnostic Equipment (Geréb and Co., Ltd., Budapest, Hungary) with a piezoelectric sensor (Micro-100S from Physical Acoustic Corporation, Princeton Junction, NJ, USA) was used. The cover of the original sample holder assembly was removed from the DSC and was replaced by a custom-made cover, which enables the sensor to be coupled to the top side of the sample. In order to allow thermal separation of the sensor from the sample a pointed steel waveguide was used (see the details and Figure 1 in [28]). The analog-to-digital converter sampling rate of the AED 404 was 8 MHz and the setup had a band-pass from 30 KHz to 1 MHz, adjusted to the frequency range of the sensor. A 30 dB preamplifier and a logarithmic main amplifier with 90 dB dynamic range were used. The threshold level was 36.5 dB, which was determined from a test measurement carried out in martensitic state without heating/cooling, when the low-level acoustic emission should not belong to the phase transformation. For the computation of the exponents, logarithmic data binning was used.

## 3. Results

**Figure 1:**
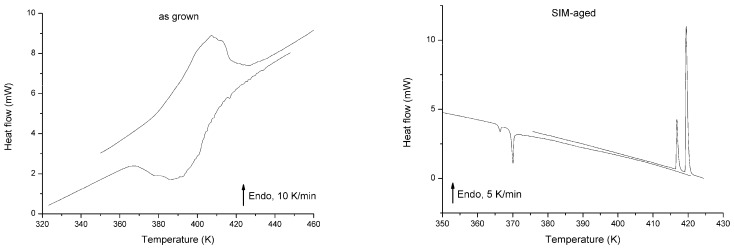
Differential scanning calorimetry (DSC) runs for the as grown (left) and stress-induced martensite stabilization (SIM)-aged (right) Ni_53_Mn_25_Ga_22_ single crystals.

**Figure 2:**
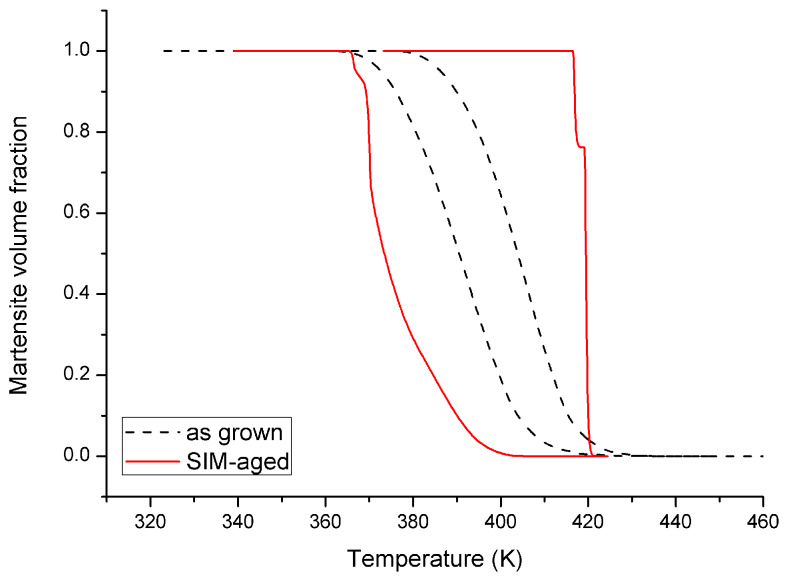
Hysteresis loops calculated from the DSC curves for the as grown and SIM-aged single crystals. The heating rates were 10 K/min and 5 K/min for the as grown and the SIM-aged samples, respectively.

The results of the DSC measurements are shown in Figure 1 for heating and cooling of the as grown and SIM-aged samples, respectively. Figure 2 shows the hysteresis loops, constructed from the measured DSC curves. The martensite volume fraction was calculated using the normalized partial integrals of the dQ/T curves [26,27] (Q is the heat of transformation, T is the temperature) from the measured DSC according to the usual procedure [27]. The values of the transformation entropy were also determined from the integrals of the dQ/T curves. Table 1 shows the austenite and martensite start and finish temperatures (A_s_, A_f_, M_s_, M_f_, respectively) and the transformation entropies. It can be seen that, although the area of the hysteresis became wider by about a factor of three, the forward and reverse transitions became sharper in the SIM-aged sample. In addition, for the SIM-aged sample the DSC curves split into two separate peaks, illustrating a more stepwise character of the transformation. Furthermore the transformation entropy decreased by about 36% due to SIM-aging.

It is worth noting that an envelope type DSC curve (like the curves for the as grown sample) can be split into small individual peaks due to different reasons such as low cooling/heating rates [19,20,26,28,29], small investigated masses [26,28], or surface roughening [26]. In our case the appearance of the two sharp peaks is rather related to the sudden jump of the martensite/austenite interface, like in [15].

**Figure 3:**
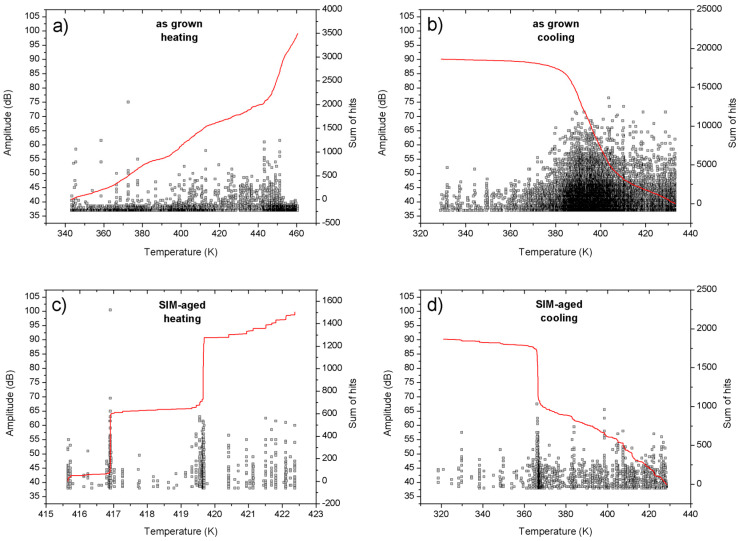
Acoustic emission hits during the transformation: heating and cooling for the quenched, (**a**,**b**) and SIM-aged (**c**,**d**) crystals. Each point represents an acoustic emission (AE) event where the amplitude and the continuous line, indicating the transition, is the cumulative number of them.

The measured acoustic noises for the as grown and SIM-aged samples are shown in Figure 3. It can be seen in Figure 1 and Figure 3, as well as in Table 1, that the value of *A_f_*–*A_s_* is definitely smaller for the SIM-aged sample, indicating a sharper martensite to austenite transition. Table 2 contains the average peak energy per one AE event for heating and cooling. The energy of an individual acoustic event, $E_{i}$, was calculated from the integral of the square of the voltage signal of the sensor, U(t): $E_{i}=\frac{1}{R}\int_{t_{i}}^{t_{i}+D_{i}}U^{2}\left(t\right)dt$, where $t_{i}$ and $D_{i}$ are the detection time and duration time of the i-th event [15,25,28] (using V and s units, as well as taking an arbitrary value for the input resistance of the amplifier as R = 1 MΩ). The probability distribution functions of the energies of the AE events usually follow damped power law behaviour [17,30,31]:

(1) $$P\left(E\right)\propto \;E^{-\varepsilon }exp\left(-E/E_{c}\right),$$

where *E* is the peak energy of an acoustic event, *ε* is the characteristic exponent, and *E_c_* is the cutoff value, which is more often much higher than the maximal AE event energy detected, and in these cases the exponential factor can be taken to be unity.

**Figure 4:**
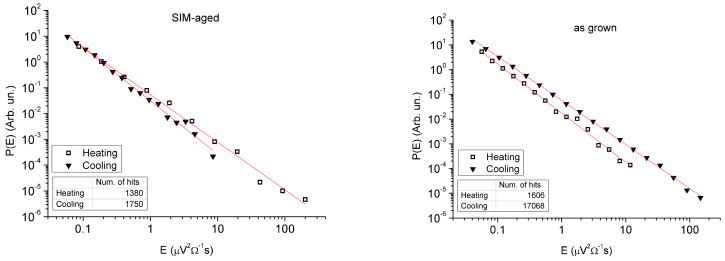
Energy distribution functions for heating and cooling in the case of the as grown (left) and SIM-aged samples (right).

Figure 4 shows the energy distribution functions during heating and cooling for the as grown and SIM-aged samples, respectively. The values of the energy exponents are also included in Table 2. It can be seen that these functions can be well approximated by a straight line over 3–4 orders of magnitude on the energy scale and we can take that $exp\left(-E/E_{c}\right)≅1$, i.e., in our measurements the number hits at higher energies became very low before the cutoff effect would become visible. It can be also seen that the exponents differ slightly but distinctly for heating and cooling in both samples. Interestingly, their deviation has a different sign: while the relative change, *γ_ε_* = (*ε_h_* − *ε_c_*)/*ε_c_*, is positive (*γ_ε_* = 0.11) for the as grown sample, its value is negative (*γ_ε_* = −0.11) after SIM-aging.

## 4. Discussion

**Figure 5:**
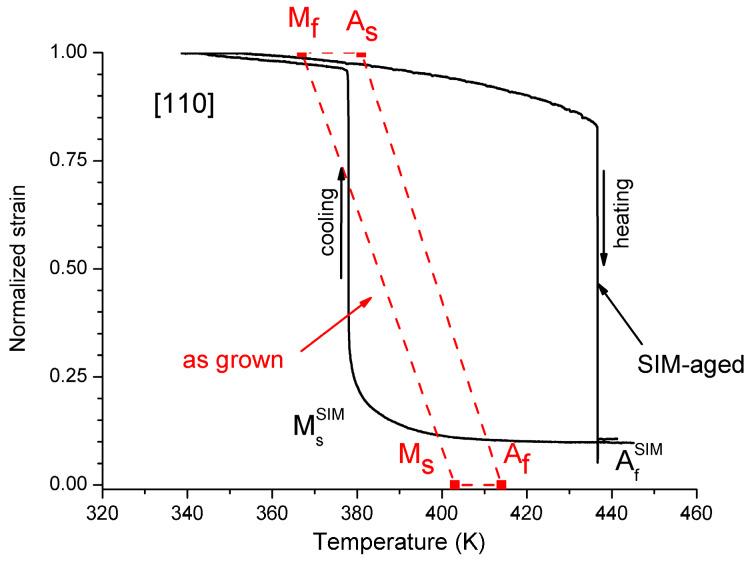
Normalized *ε* versus *T* hysteresis loops for the as grown and SIM-aged samples oriented [110]_L21_ direction. The dashed lines for the as grown sample illustrate schematically the hysteresis loop based only on the start and finish temperatures determined by DSC in [5] and [22] (|ε_tr_| = 5.4 %).

Our results for the hysteresis loops can be compared with the results of [5] and [22], where the transformation strain versus temperature (*ε*~*T*) after SIM-aging along [110] and [123] direction was carried out and the start and finish temperatures were determined from DSC runs in the as grown state of the same single crystal (Figure 5). Comparing Figure 3 and Figure 5 we can conclude that the agreement is reasonable. It is worth noting that according to [5] and [22] the shapes and the positions of the hysteresis loops after SIM-aging are very sensitive to the orientation of the uniaxial stress applied during SIM-aging and also to the twinning/detwinning of the martensite variant [5,6,22].

Thus, we can conclude that SIM-aging, the stabilization of detwinned martensite [22], in our case caused a significant increase of the area of the hysteresis and there is practically no shift of the hysteresis. Furthermore, the transitions became sharper both for cooling and heating, especially the heating, indicating a burst-like transition.

**Figure 6:**
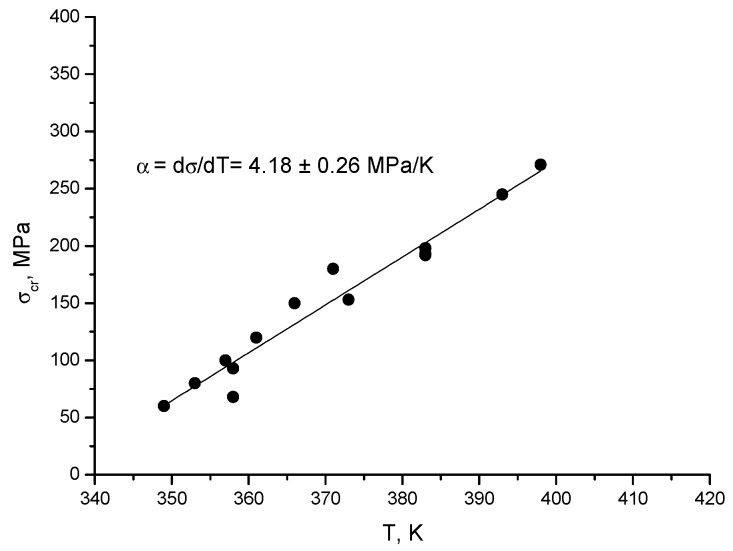
Temperature dependence of the critical stress for [110]_L21_-oriented Ni_53_Mn_25_Ga_22_ single crystal in compression before SIM-aging.

The values of the transformation entropy shown in Table 1 can be compared with the value published in [32]: the value obtained for the as grown sample is in good agreement with the value belonging to a similar composition of our sample. On the other hand, the transformation entropy can also be estimated from the Clausius–Clapeyron relation for the temperature dependence of the critical stress, *σ_cr_*:

(2) $$\frac{\partial \sigma_{cr}}{\partial T}=-\frac{\Delta s}{V\varepsilon_{tr}},$$

where *V* = 7.5 cm^3^/mol is the molar volume and *ε_tr_* is the transformation strain. For a sample of the same composition and SIM-aged along the <123> axis, *dσ_cr_*/*dT* = 4.4 MPa/K was obtained [5], and using this with *ε_tr_* = 4.2% [22], we have −Δ*s* = 1.5 J/mol∙K. On the other hand, for [110]-oriented crystal before SIM-aging, *dσ_cr_*/*dT* = 4.2 MPa/K and |ε_tr_| = 5.4% is obtained (see Figure 6), and thus, −Δ*s* = 1.1 J/mol∙K. These values are also in reasonable agreement with the values given in Table 1.

Our results in Table 1 confirm the conclusions of [15] and [24]: the transformation entropy decreased by about 36% after SIM-aging. This value is in very good agreement with the value found in [15]: 36%, but less than the 12% decrease found in a Cu-based shape memory alloy [24]. Thus, further measurements are desired to decide whether this difference is due to the special SIM-aging in ferromagnetic shape memory alloys and whether Cu-based alloys form a different sub-class or not. Indeed, in ferromagnetic shape memory alloys there is a magnetic contribution to the transformation entropy (see, e.g., [32,33,34]) and a possible contribution of this to the change of Δ*s* after SIM-aging calls for further investigation.

It is clear that the width of the hysteresis increased for the SIM-aged sample by a factor of three. This, together with the practically non zero shift of the hysteresis, is a bit surprising in light of the expectations that such SIM-aging can decrease the dissipative energy and shift the equilibrium transformation temperature to higher values (see, e.g., [6]). Taking into account that the transformation entropy decreased only by about 36%, the increase of the area of the hysteresis loop by about a factor of 3 cannot be attributed only to the entropy change (Δ*T*, the width of the hysteresis, is inversely proportional to −Δ*s*). Thus, some additional microscopic processes should have a contribution to this, and the results of the AE measurements can give some additional hints in this respect. Indeed, there are two interesting features of such measurements (see Table 2 and Figure 3 and Figure 4):

(i) There are two sudden jumps on the plot of the cumulative number of the acoustic emission events versus temperature for heating (Figure 3c), correlating very well with the two sharp peaks on the corresponding DSC curve (Figure 1).
(ii) The energy distributions of the acoustic hits showed nice power law behaviour and the energy exponents were different for heating and cooling; this asymmetry had different signs for the as grown and SIM-aged samples.

These findings indicate that some microscopic details of the forward and reverse transformations are different in the SIM-aged sample. Indeed, before SIM-aging, the self-accommodation multivariant structure of the L1_0_ martensite forms at the cooling-heating cycle, while in the SIM-aged crystal the oriented detwinned variant of martensite grows during the stress-free cooling-heating cycle [5,22].

It is known that the energies of the individual acoustic events are directly related to the relaxation (and dissipation) of the energy of the elastic waves emitted during an individual jump of the moving austenite/martensite interface [17,30]. The plot of the cumulative number of AE events during heating (for which the transition indeed has a burst-like character) clearly correlates with the two separate DSC peaks (statement i)).

In addition, the two samples showed a different type of asymmetry of martensitic transformation (ii)): for the as grown state the asymmetry was positive (*γ_ε_* = (*ε_h_* − *ε_c_*)/*ε* = 0.11), while in the case of the SIM-aged sample the asymmetry was negative (*γ_ε_* = −0.11). It was shown in [35] that for the interpretation of deviations from the symmetric behaviour (*γ_ε_* = 0) it had to be assumed that the thermoelastic balance condition was not fulfilled, i.e., the stored elastic energy during cooling should be different from the negative of the released elastic energy during heating, since different parts of the elastic energy are relaxed by AE during cooling and heating. In [35] it was additionally supposed that the frictional-type dissipative events and nucleation effects were the same in both directions, which was in contrast to [35], where the role of nucleation effects were emphasized. Thus, it was concluded in [35] that if the relaxed fraction of the total elastic strain energy, *E*^↓^*_t_*, which would be stored without relaxations, during cooling, *β*^↓^ = *E*^↓^*_rt_*/*E*^↓^*_t_*, is larger than the corresponding relaxed fraction during heating, *β*^↑^(*1* − *β*^↓^) = *E*^↑^*_rt_*/*E*^↑^*_t_* ($\beta^{↑}=\frac{E_{rt}^{↑}}{E_{t}\left(1-\beta^{↓}\right)}$, since only *E_t_*(*1* − *β*^↓^) elastic energy has been stored in the martensitic state), then the asymmetry is positive (i.e., if $\frac{E_{rt}^{↑}}{E_{rt}^{↓}}<1$, or $\beta^{↑}<\frac{\beta^{↓}}{\left(1-\beta^{↓}\right)}$). Similarly, it was also shown in [35] that for positive asymmetry the relative change of the number of events (or acoustic energies) is negative. Indeed, it can be seen in Table 2 that the *N_h_/N_c_* ratio is larger than unity for heating and smaller than unity for cooling. Thus, for the interpretation of the positive as well as negative asymmetry observed, one has to go beyond the thermoelastic balance assumption. Of course, such considerations call for detailed thermodynamic analysis as well as microscopic observations of the details of the martensitic transformations in the as grown and SIM-aged samples. Nevertheless, we can provide similar qualitative arguments. The negative asymmetry observed for the SIM-aged sample means that during the development of the single variant martensitic structure the relaxation of the elastic energy in the form of AE should be less than for heating, while the opposite statement should be valid for the as grown sample. If we take into account that for the transformation of the martensite stabilized structure in the SIM-aged sample the plausible initial state is the martensite, while in the as grown sample the austenite state is the stable one, then from the point of view of the elastic energy accumulation and release, the cooling and heating process can be viewed if inverted, and thus, a change in the sign of the asymmetry is also expected.

## 5. Conclusions

- The forward (from austenite to martensite) and reverse transitions became sharper, and the width of the hysteresis increased in the SIM-aged sample. On the other hand, in spite of the expectations, the SIM-aging did not cause a shift of transformation temperatures to higher values.
- The transformation entropy is smaller for the SIM-aged sample. Taking into account that it decreased only by about 36%, the increase of the area of the hysteresis loop by about a factor of 3 cannot be attributed solely to the entropy change (Δ*T*, the width of the hysteresis, is inversely proportional to −Δ*s*).
- The two sudden jumps on the plot of the cumulative number of the acoustic emission events versus temperature for heating (Figure 3c) correlate very well with the two sharp peaks on the corresponding DSC curve (Figure 1).
- The energy distributions of acoustic emission hits showed power law behavior. The SIM-aging changed the sign of the asymmetry of the power exponents characterising the energy distributions: the relative change was positive (11%) for the as grown sample, and its value was negative (−11%) after SIM-aging. This can be interpreted with the failure of the thermodynamic balance: the stored elastic energy during cooling should be different from the released elastic energy during heating.

## Figures and Tables

**Table 1:** Transition temperatures before and after SIM-aging and absolute values of the transformation entropy, Δ*s*, derived from DSC measurements. The error bars are about ± 3 K for the start and finish temperatures. The entropy values are averaged for heating and cooling and the error bar is about ± 0.08 J/mol·K. Obviously the transformation entropy is negative for cooling.

Sample	A_s_ (K)	A_f_ (K)	M_s_ (K)	M_f_ (K)	Δ*s* (J/mol·K)
as grown	378	426	415	365	1.37
SIM-aged	416	420	396	366	1.01

**Table 2:** Critical energy exponents, *ε*, the total numbers of hits, *N_total_*, the number of hits per unit mass, *N/m*, and the average peak energy per one event (Eav=∑iEiN , where *E_t_* is the sum of peak energies, $\sum_{i}E_{i}$, per unit mass).

Sample	AE for Heating				AE for Cooling			
	*N_total_*	*N/m* (1/mg)	*E_av_* (Arb. Units)	*ε*	*N_total_*	*N/m* (1/mg)	*E_av_* (Arb. Units)	*ε*
as grown	1606	36	0.436	1.98 ± 0.05	17068	385	1.18	1.78 ± 0.05
SIM-aged	1380	97	0.634	1.84 ± 0.05	1750	123	0.257	2.06 ± 0.05
